# Colorectal Cancer and Alcohol Consumption—Populations to Molecules

**DOI:** 10.3390/cancers10020038

**Published:** 2018-01-30

**Authors:** Marco Rossi, Muhammad Jahanzaib Anwar, Ahmad Usman, Ali Keshavarzian, Faraz Bishehsari

**Affiliations:** Division of Digestive Diseases, Hepatology, and Nutrition, Department of Internal Medicine, Rush University Medical Center, Chicago, IL 60612, USA

**Keywords:** alcohol, CRC, polyposis, metabolism, epigenetics, immunity

## Abstract

Colorectal cancer (CRC) is a major cause of morbidity and mortality, being the third most common cancer diagnosed in both men and women in the world. Several environmental and habitual factors have been associated with the CRC risk. Alcohol intake, a common and rising habit of modern society, is one of the major risk factors for development of CRC. Here, we will summarize the evidence linking alcohol with colon carcinogenesis and possible underlying mechanisms. Some epidemiologic studies suggest that even moderate drinking increases the CRC risk. Metabolism of alcohol involves ethanol conversion to its metabolites that could exert carcinogenic effects in the colon. Production of ethanol metabolites can be affected by the colon microbiota, another recently recognized mediating factor to colon carcinogenesis. The generation of acetaldehyde and alcohol’s other metabolites leads to activation of cancer promoting cascades, such as DNA-adduct formation, oxidative stress and lipid peroxidation, epigenetic alterations, epithelial barrier dysfunction, and immune modulatory effects. Not only does alcohol induce its toxic effect through carcinogenic metabolites, but alcoholics themselves are predisposed to a poor diet, low in folate and fiber, and circadian disruption, which could further augment alcohol-induced colon carcinogenesis.

## 1. Introduction

Colorectal cancer (CRC) is the third most commonly diagnosed cancer among men, and the second most diagnosed among women worldwide [1]. It also constitutes a significant proportion of the global burden of cancer morbidity and mortality, being the 4th most common cause of cancer mortality, and accounting for 600,000 deaths annually [1,2]. With its relatively high incidence, CRC remains one of the major areas of healthcare spending, with the mean cost of disease management in the first year of diagnosis being around $32,648 [3]. The occurrence of CRC occurrence is closely linked to the environment and Western lifestyle [4]. Therefore, recognizing and addressing environmental factors contributing to the disease development and progression could reduce the CRC burden [4]. Alcohol consumption, a lifestyle habit commonly associated with Western lifestyle, is a major contributor to colon carcinogenesis [5]. Given the high prevalence of alcohol drinking and high incidence of CRC worldwide, the contribution of alcohol consumption toward the development of CRC, particularly in the developed countries, constitutes a significant proportion of the condition’s global burden [6]. It is estimated that alcohol increases the chance of CRC by more than 50% [7]. Alcohol has numerous direct and indirect effects that contribute toward carcinogenesis [8,9,10]. Its metabolism can be either oxidative or non-oxidative, producing acetaldehyde as well as a number of other metabolites [11]. Alcohol, along with its metabolites, can promote carcinogenesis through numerous mechanisms [8,9,12]. These mechanisms cause genetic, epigenetic, biochemical, and immunological abnormalities, leading to chronic inflammation and cancer formation [5,9,10,13]. Here, we highlight the most updated epidemiological evidence studies on alcohol and the risk of CRC. We will then review the mechanisms by which chronic alcohol intake may lead to CRC formation. Understanding mechanisms underlying the alcohol induced CRC will provide us with opportunities to identify factors/pathways that promote the alcohol’s effect. This would help us identify alcohol drinkers at risk for CRC formation, develop personalized preventive strategies, and finally, discover therapeutic interventions targeting pathways responsible for the increased susceptibility to carcinogenesis effects of alcohol.

## 2. The Epidemiology of Alcohol and Colon Carcinogenesis

### 2.1. Alcohol and CRC

Alcohol intake, even in small amounts, has been proposed to be associated with an increased risk of CRC [14,15,16]. The association of alcohol consumption and CRC risk is dose-dependent [14,17,18]. CRC risk is particularly remarkable with heavy drinking, while data on the risk of CRC due to light to moderate drinking is inconsistent [14,17,18]. A recent meta-analysis of literature from 1966 to 2013 shows an overall relative risk (RR) of 1.21 (95% CI, 1.01–1.46) for subjects who drink 56.5 g/day [14]. Similarly, another meta-analysis focusing on cohort studies from North America and Europe found that subjects consuming ≥50 g/day had an overall CRC RR of 1.52 (95% CI, 1.27–1.81) [16]. Although data on binge alcohol consumption and CRC risk is limited, mechanistically, this is possible, as binge drinking has been shown to increase CYP2E1 and HIF-1α levels in human samples [19]. In addition to CRC risk, one German study reported on an increased CRC mortality from heavy drinking (>50 g/day for men, >25 g/day for women, HR = 1.37, 95% CI, 1.06–1.78, *p* = 0.015) [20], though these findings were not replicated by another recent large study [21]. Nonetheless, a meta-analysis supported an association between heavy alcohol drinking (≥50 g/day of ethanol) and CRC mortality [14]. Several mechanisms have been proposed to explain the alcohol effect on cancer mortality, which includes effects of alcohol on tumor growth via insulin signaling, selection for aggressive clones, impairment of host defenses, poor diet, and patient noncompliance [22,23,24,25].

Other factors can modulate alcohol’s effects on CRC risk [26]. One such factor is a family history of the disease [26]. One study found that, with an alcohol consumption of ≥30 g/day, the RR of CRC significantly increased from 1.20 to 2.80 when a family history of CRC was present [26]. Another modulatory factor could be sex; among subjects that drink 12.5–50 g/day, males have a CRC RR of 1.10 (95% CI, 1.03–1.18) while female subjects have a RR of 0.87 (95% CI, 0.65–1.16) [14]. Body mass index (BMI) and weight could also interact with alcohol on CRC risk. A Canadian study reported that, for subjects who consumed an alcohol beverage at least once a week for 6 months or longer, those with a BMI < 30 had an overall CRC OR of 0.8 (95% CI, 0.60–1.10) [27]. But, those with a BMI > 30 had an overall CRC OR of 2.2 (95% CI, 1.20–4.00, *p*_trend_ < 0.05) [27]. A fourth factor is race, especially with regard to CRC screening [28,29]. In New Mexico, one study found a significant difference in the CRC screening of Hispanic females (ages ≥ 50 years) who were at risk of drinking more than one alcoholic drink a day compared to Caucasian females of the same age and alcohol risk (*p* < 0.01, Chi-square analysis) [29]. Despite the known interaction of smoking and alcohol in malignancies of upper gastrointestinal tract, there is little evidence that smoking modulates the risk of alcohol-induced CRC. Therefore, although smoking is a major CRC risk factor, a connection with alcohol cannot currently be made [30].

Alcohol’s influence could also vary based on individual differences in alcohol metabolisms. Alcohol’s impact on CRC has been widely implicated in Asian populations, due to the prevalence of genetic polymorphisms in enzymes involved in alcohol metabolism [31,32,33]. For example, ADH1B polymorphisms have been associated with an increased risk of CRC [34]. In a recent meta-analysis, the ADH1B Arg47His polymorphism was determined to have a CRC OR of 1.18 (95% CI, 1.01–1.36, *p* = 0.03) [34]. Besides those associated with ADH1B, associations between CRC risk and other polymorphisms commonly found in Asian populations, like those associated with ALDH2, have been inconsistent [35].

Alcohol consumption may not equally affect all areas of the colorectum [36,37]. Multiple studies show that, for high alcohol consumption, the highest cancer hazard ratio (HR) resides in the rectum [36,37]. One such study found that, for an average lifetime consumption rate of >60 g/day, the HR = 2.59 (95% CI, 1.62–4.13) for the rectum, HR = 2.22 (95% CI, 1.20–4.13) for the distal colon, and HR = 1.22 (95% CI, 0.59–2.51) for the proximal colon [37]. This trend of the HR being the highest in the rectum, lower in the distal colon, and the lowest in the proximal colon, was reproduced in a Dutch study for subjects that consume ≥30 g/day [36].

### 2.2. Alcohol and Polyps

CRC rises from pre-cancerous neoplastic lesions in the colon, called polyps. Several studies support a positive association between alcohol consumption and the risk of adenomatous polyp formation [38,39,40,41]. A recent meta-analysis showed an increased risk of adenoma, even with light to moderate drinking [42]. The association of alcohol intake with colonic polyps could be particularly strong for larger adenomas [40]. Similar to CRC, the link of alcohol and polyp risk could be dose dependent; consumption of seven or more drinks/weeks increased risk of adenoma formation by odds ratio of 2.04 (95% CI, 1.28–3.26) [38]. Besides adenomatous polyps, alcohol has been recently shown to increase the risk of formation of serrated polyps as well [43]. Serrated polyps are less common than conventional adenomas, and have been recognized as the alternative pathway to CRC [43,44]. The relative risk of serrated polyp formation is 1.30 (95% CI, 1.15–1.48), when comparing drinkers that drink high vs low amounts of alcohol [43]. The effects of alcohol on polyp formation could be possibly modified by other demographic factors, including a history of previous adenomas [45]. For example, among heavy drinkers that consumed >50 g alcohol/day, the percentage of cases that developed a high-risk adenoma or CRC was 72% when those heavy drinkers had at least one previous high-risk adenoma or cancer; there was a 57% development of these conditions without a prior high-risk adenoma or cancer (*p* < 0.01, global χ^2^ test) [45]. The association of alcohol intake with colonic polyps propose an association of colonic polyps with alcoholic liver disease (ALD), a well-established sequela from alcohol [46]. In a Canadian study, 80 orthotopic liver transplant (OLT) patients were screened for polyps before and after the transplant [46]. It was found that those with a history of ALD were 11 times more likely to develop post-transplant polyps in the colon (OR = 11.3, 95% CI 3.2–39.4, *p* < 0.001) [46]. We could not find any studies that directly test association of binge alcohol consumption and the CRC risk. Such an association seems likely, given that binge alcohol consumption could result in ALD, and that ALD is an established risk factor for colonic neoplasm.

Overall, most studies agree on recognizing chronic alcohol consumption as a risk factor for colorectal polyposis and cancer formation, suggesting the contribution of alcohol to colorectal carcinogenesis [8,9,12]. The risk is even stronger when combined with other risk factors, like obesity [27,38,47,48,49]. In the following section, we will discuss possible molecular mechanisms that could mediate alcohol’s effects on CRC.

## 3. The Metabolism of Ethanol

### 3.1. General Metabolism

**Figure 1:**
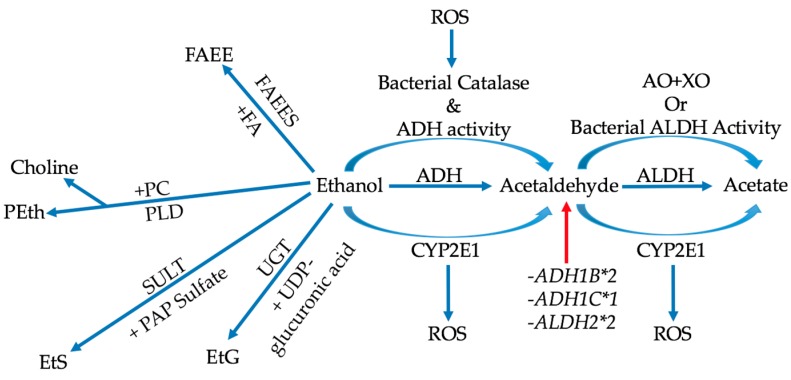
The majority of ethanol metabolism is oxidative where carcinogenic acetaldehyde is produced via alcohol dehydrogenase (ADH), CYP2E1, or bacterial catalases. The acetaldehyde is reduced by being converted to acetic acid by aldehyde dehydrogenase (ALDH), CYP2E1, or the combination of aldehyde oxidase (AO) and xanthine oxidase (XO). Conversion with CYP2E1 tends to produce reactive oxygen species (ROS), adding to carcinogenesis. Polymorphisms in the ADH and ALDH enzymes increase acetaldehyde concentrations (red arrow). Non-oxidative metabolism leads to the production of metabolites using various types of molecules. In the case of nucleotides, EtG and EtS are produced. For lipids, PEth and FAEE are produced. EtS = ethyl sulfate; SULT = sulfotransferase; PAP Sulfate = 3′-phosphoadenosine-5′-phospho sulfate; PEth = phosphatidylethanol; PC = phosphatidylcholine; PLD = phospholipase D.

The overall metabolic processes of ethanol can be subdivided into two main categories: oxidative and non-oxidative metabolism (Figure 1) [11]. The majority of ethanol metabolism consists of oxidative metabolism, in which ethanol is converted to acetaldehyde either by alcohol dehydrogenase (ADH), cytochrome P450 2E1 (CYP2E1), or bacterial catalase [11,50,51,52]. Acetaldehyde is then converted into acetic acid via aldehyde dehydrogenase (ALDH), CYP2E1, or the combination of aldehyde oxidase (AO) and xanthine oxidase (XO) [11,50,53]. It must be noted that metabolism through CYP2E1 is a large producer of reactive oxygen species (ROS), which can affect many mechanisms [11,12]. Of ethanol’s metabolites, acetaldehyde is widely considered to be the most potent in colorectal carcinogenesis through its interactions with various cellular and biochemical processes [10,12,51,53,54,55]. Non-oxidative metabolism, although occurring at lower rates, has a range of physiological influence that can be quite profound due to the wide variety of metabolites produced [11,56]. This variety is derived from the ability of ethanol to “insert” itself into the structures of significant biochemical molecules [56]. For example, ethanol can undergo an enzymatic esterification with fatty acids (FA) to produce fatty acid ethyl esters (FAEE) via FAEE synthase (FAEES) [56]. Another example is the enzymatic production of ethyl glucuronide (EtG) from uridine 5′-diphospho-glucuronic acid (UGT) and ethanol [56].

### 3.2. Enzyme Polymorphisms and Bacterial Metabolism

Metabolic variations in production or degradation of alcohol metabolites could affect their availability and, as such, modulate alcohol effect on the tissue [8,54,57]. Genetic polymorphisms, like ADH1B*2 and ADH1C*1, increase the activity of alcohol dehydrogenase and, thus, increase the amount of acetaldehyde present [11,54]. On the other hand, two ALDH2*2 alleles can render the aldehyde dehydrogenase enzyme nearly inactive, preventing acetaldehyde’s degradation [8]. Besides genetic variations, bacterial metabolism of ethanol also increases the amount of acetaldehyde formed in the colon [11,51,58,59]. Bacterial catalase can produce acetaldehyde from ethanol like alcohol dehydrogenase [58,60,61]. Certain bacteria in the colon, such as the *Enterobacteriaceae*, also have ADH and ALDH activities [62]. However, bacterial ALDH activities tend to be less than their ADH activities, allowing colonic acetaldehyde to accumulate as ethanol consumption increases [59,62,63,64]. Acetaldehyde production by anaerobic bacteria in the colon, such as *Ruminococcus* and *Bifidobacterium*, can also increase upon exposure to ROS, resulting in a vicious cycle of accumulating carcinogens [59]. With these bacterial production methods present, local acetaldehyde concentrations in the colon increase past the minimum mutagenic concentration (MMC), and greatly contribute to carcinogenesis [58,59,60,61].

## 4. Genetic Stability, Epigenetic Modifications, and Mutation

### 4.1. Direct DNA Damage

There are quite a few ways in which ethanol and its metabolites can affect genetic stability and expression that may lead to colorectal carcinogenesis [10,12,55,65,66,67]. Alcohol metabolites can directly affect DNA stability [12,68]. Acetaldehyde can bind to deoxynucleotides to form DNA adducts [12]. In addition, malondialdehyde (MDA) and 4-hydroxynoneal (4-NHE), metabolites from ROS-induced lipid peroxidation, can also form DNA adducts [12,55]. Thus, metabolic processes increasing ROS formation can also instigate this direct DNA damage and encourage colorectal carcinogenesis. In fact, in a study of 46 patients, those that were alcoholics formed more ethano-DNA adducts than the non-drinking controls [69]. In this study, the correlation between the formation of these adducts and colorectal cancer formation was made, as hyperproliferation of the colonic mucosa was observed [69]. Besides direct DNA damage, there are also indirect modalities through which ethanol can disturb genomic integrity [10,12,55,65,66,67].

### 4.2. Indirect DNA Instability

**Figure 2:**
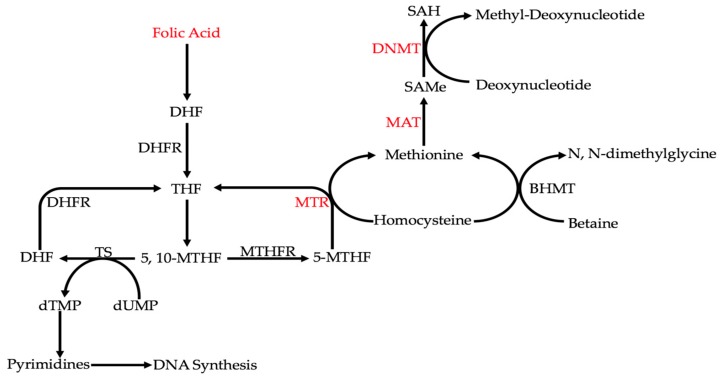
Ethanol indirectly interferes with DNA integrity by affecting key points (labeled in red) or enzymes in folate metabolism. From these key points, DNA synthesis and methylation are hindered. A reduction in folic acid levels decreases all reactions in the above pathway. Reducing methionine synthase (MTR) activity inhibits both DNA synthesis and DNA methylation. Reducing methionine synthase (MAT) and DNA methyltransferase (DNMT) activities inhibits DNA methylation.

Ethanol metabolism can affect colorectal carcinogenesis by influencing single-carbon metabolism (Figure 2) [10,70,71]. Under normal circumstances, homocysteine is converted to methionine through either methionine synthase (MTR) or betaine homocysteine methyltransferase (BHMT) [10]. That methionine is converted to *S*-adenosylmethionine (SAMe) by methionine adenosyl transferase (MAT) [10]. In turn, methyl groups from SAMe are used by DNA methyltransferases (DNMTs) to methylate deoxynucleotides [10]. MTR, MAT, and DNMTs are some of the key enzymes in single-carbon metabolism that regulate DNA methylation [10]. Ethanol and its metabolites can affect the activities of all of these enzymes [10]. It was found that the activities of these enzymes are reduced with alcohol and/or acetaldehyde [10]. Through the inhibition of these enzymes, ethanol metabolism decreases DNA methylation and dysregulates epigenetic patterns [10]. This overall decrease in DNA methylation, or global DNA hypomethylation, has been widely shown in CRC [70,71]. For example, hypomethylation of long interspersed nuclear element-1 (LINE-1) sequences in the CRC tissues of 77 patients was shown to increase *MET* expression in metastases [71]. To further emphasize the significance of global DNA hypomethylation in CRC, curcumin, an anti-cancerous agent, induces DNA methylation in the HCT116, HT29, and RKO cell lines [70].

Another way that alcohol can indirectly affect genetic expression in CRC is by interfering with folate metabolism [10]. Folate is converted into tetrahydrofolate (THF), and then into 5,10-methylenetetrahydrofolate (5,10-MTHF) [10]. At this point, 5,10-MTHF can be used to synthesize DNA by participating in the conversion of dTMP from dUMP via thymidylate synthase (TS) [10]. 5,10-MTHF can also be converted to 5-methyltetrahydrofolate (5-MTHF) by methylenetetrahydrofolate reductase (MTHFR), which is used to produce methionine and, by extension, *S*-adenosylmethionine (SAMe) [10]. It must be noted that the MTHFR polymorphisms C667T and A1298C, as well as the TS polymorphism TS1494del6, have been found in cases with alcohol consumption [10,72]. It has been shown that acetaldehyde can degrade folate [52]. Therefore, by interfering with folate metabolism, both DNA integrity and methylation are affected [10]. Interfering with DNA synthesis can cause double-strand breaks [10]. A reduction in methionine synthesis would lead to global DNA hypomethylation [10]. In the case of CRC, along with a reduction in folate, significantly increased uracil misincorporation and DNA hypomethylation, which were observed in samples of polyps and the surrounding tissues [73].

Alcohol intake can affect other cofactor nutrients, like vitamins B6 and B12, as well [74]. Vitamin B6 is the cofactor of serine hydroxymethyltransferase (SHMT) and cystathionine β-synthase (CBS) [74]. Vitamin B12 is the cofactor of MTR [74]. Thus, these vitamins are important in both the formation and metabolism of THF, 5,10-MTHF, and methionine [74]. Because chronic alcohol consumption can result in malnutrition and deficiencies in these vitamins, DNA synthesis and methylation would be affected as a result [74]. It must be noted, however, that the levels of these vitamins can vary depending on the individual conditions and circumstances [74]. This uncertainty is reflected in the literature, as a cohort study of subjects from the Nurses’ Health Study (NHS) did not show a significant correlation between levels of B vitamins, alcohol intake, and CRC risk with respect to the status of *BRAF* mutation, a common pathway involved in colon carcinogenesis [75]. However, despite these results, a lack of B vitamins has been widely implicated in colon carcinogenesis overall [10].

In the context of alcohol and colon carcinogenesis, the effects on DNA repair mechanisms are controversial [34,76,77,78,79]. Both nucleotide excision repair (NER) and base excision repair (BER) have been proposed to be involved in correcting alcohol-induced DNA damage [79,80]. NER repairs DNA by cleaving parts containing erroneous bases and sequences [79]. This cleaving is accomplished by first recognizing the erroneous bases using a protein complex to unwind the DNA, followed by damage recognition and formation of a preincision complex involving proteins such as XPA, RPA, XPG, and ERCC1. Upon removal of the damaged piece of DNA, repair DNA synthesis is performed by DNA polymerases [81]. BER works by a different mechanism, as it results in the replacement of single, chemically modified nucleotides instead of a sequence [82], first by recognizing the erroneous, chemically modified bases and cleaving them from the DNA strand using glycosylases, such as SMUG1, OGG1, and APE1 [82]. Other than glycosylases that remove chemically-modified bases, there are also enzymes that remove the specific chemical modifications to such bases [77]. For example, MGMT removes alkyl groups from guanine [77].

As previously stated, it is plausible that the alcohol-induced DNA damage and risk of CRC vary based on the capacity of these DNA repair mechanisms [34,76,77,78,79]. In a group of Chinese Han colon cancer and control subjects, there seemed to be no association between alcohol consumption and mutation in *APE1* [76]. Similarly, in the same group of subjects, insignificance was also found between alcohol consumption and mutation in the genes *XRCC1* and *OGG1* [76]. Insignificance to alcohol consumption in CRC was observed for mutations in NER proteins like XPA A23G, XPC Lys939Gln, XPD Lys751Gln, and XPD Asp312Asn in a group of 397 Danish colon cancer patients compared to 800 Danish control patients [78]. Because these NER proteins play a major role in CRC, more work needs to be done in order to fully elucidate the effects of alcohol consumption on the range of mutations that they can have [79,80,83]. Significant associations have been made between alcohol intake and mutation in *MGMT* in the presence of the *Kras* mutation in colon cancer [77].

## 5. Non-Coding RNAs, Cell Signaling, and Stemness

### 5.1. Non-Coding RNAs and Their Effects

Ethanol and its metabolites can also affect gene expression in CRC by altering the levels of certain miRNAs (microRNAs) [9,84]. By changing the relative amounts of certain miRNAs, ethanol can indirectly influence processes such as lipid metabolism, epithelial to mesenchymal transition (EMT), angiogenesis, and the immune response, affecting carcinogenesis [9,85,86]. Some examples of miRNAs that are dysregulated with ethanol are miR-34a, miR-21, and miR-135 [87]. miR-34a has been widely known to be a tumor suppressor, directly regulated by p53 [88]. Like the other miRNAs to be discussed in this review, it has been known to take part in many processes beneficial to the carcinogenic state in the colon [85,88,89,90]. Normally, miR-34a is a regulator of hepatic glucose, lipid, and drug metabolism [90]. It is typically downregulated in cases of colorectal cancer [87]. Dysregulated miR-34a usually leads to disorders such as obesity, dyslipidemia, and fatty liver disease [91,92]. However, what is interesting about ethanol exposure is that miR-34a increases in an alcohol-fed rat model, which may seem to be preventative against carcinogenesis [9,93]. Indeed, miR-34a has been shown to interact with the TGF-β/SMAD4 pathway to reduce EMT and increase treatment susceptibility [89]. However, it was shown in a rhesus macaque model that the miR-34a promoter is hypomethylated, leading to increased hepatic miR-34a levels [94]. Such overexpressed miR-34a can, eventually, lead to immune dysregulation and inflammation [92]. Therefore, the unusual dysregulation of miR-34a can either negatively or positively regulate the carcinogenic state through its multiple functions.

Ethanol intake affects other miRNAs, including miR-21, involved in colon carcinogenesis [9,84]. miR-21 is an oncogenic miRNA whose overexpression has been associated with poor prognoses and lower overall survival counts in cases of CRC [95]. It has been associated with multiple cell signaling pathways, leading to oncogenic effects on immunity and stemness [85,96]. Increased miR-21 levels could be mediated via IL-6 signaling and STAT3 activation [85], and in turn, increase NF-κB and instigate inflammation [85]. miR-21 can further increase a carcinogenic immune state in the tumor microenvironment by modulating IL-10 and prostaglandin E2 (PGE2) production, and decreasing the amount of CD8+ T cells [96]. In addition, miR-21 also promotes cell survival by downregulating the extrinsic apoptotic pathway, as was shown in alcohol-treated human hepatocytes [97]. Besides the IL-6/STAT3 pathway, miR-21 is also upregulated by Ras through increases in AP1 [84]. Increased miR-21 increases β-catenin and SOX-2 expression, which leads to increased cancer cell stemness [84]. Therefore, by upregulating miR-21, ethanol could increase colon carcinogenesis through increased cancer cell stemness, cell survival, and an altered immune response [84,96,97].

miR-135 is a miRNA that decreases with ethanol exposure [9,98]. It also plays a role in colon carcinogenesis, as miR-135a and miR-135b have been shown to suppress *APC,* a key pathway commonly involved in CRC [99]. miR-135 has also been shown to be a regulator of focal adhesion kinase (FAK) [100]. FAK is a protein tyrosine kinase that compromises epithelial barrier junctions and encourages cell motility [101,102]. In fact, FAK forms a signaling axis with VEGF, VEGFR2, and AKT (known as the “VEGF–VEGFR2–AKT/FAK signaling axis”) to encourage angiogenesis [103]. Therefore, by reducing miR-135, alcohol can not only help develop CRC by upregulating APC-β-catenin pathway, but may also enhance cell motility and metastasis via increased FAK [101,102,103]. 

### 5.2. Altered Histone Modifications

Another epigenetic mechanism through which alcohol can modulate gene expression is via alterations in histone modifications [9]. Alterations in the modifications on histone 3 have been especially reported [9,104,105]. It has been shown in ethanol-treated rat hepatocytes that dimethylated H3K4 increases, while dimethylated H3K9 decreases [104]. Similarly, in hepatic samples from ethanol binged rats, phosphorylation on ser-10 and ser-28, as well as K9 acetylation on H3, were also increased [105]. The H3K9 acetylation was associated with increased phosphorylation of ERK1/2 [105]. Not only that, but H3K9’s histone acetyltransferases (HAT), p300 and GCN5, have also been shown to be affected by ethanol [106,107]. p300 was shown to increase upon ethanol treatment [106]. GCN5, when knocked down in human hepatoma cells, inhibited ethanol-induced H3K9 acetylation [107]. Finally, ethanol can affect the expression of histone deacetylases (HDACs) [108]. It was shown in an ethanol-binged mouse model that ethanol decreased the expression of HDAC 1, 7, 9, 10, and 11, and increased HDAC 3 expression [108]. SIRT-1, the HDAC that is regulated by miR-34a, decreases with ethanol [9]. Although many of these ethanol-induced modifications have been shown to be involved in the liver damage from alcohol, alterations in HDAC are repeatedly shown in CRC in general, and thus, involvement of HDACs in alcohol-induced colon carcinogenesis is quite possible [9].

### 5.3. The Hedgehog Pathway

The Hedgehog pathway is greatly influenced by ethanol [109]. This pathway has been especially relevant to conditions such as hepatocarcinoma, pancreatitis, and fetal alcohol spectrum defects (FASD) [109,110,111]. It appears that hedgehog signaling, in the case of hepatocarcinoma, is instigated by ethanol exposure, and that through this signaling, EMT is encouraged [109]. Such an increase in EMT can be connected to hedgehog’s role in stemness, as fetal alcohol exposure can lead to birth defects [111]. Alcohol’s influence on fetal hedgehog signaling has been shown to be due to deficient sonic hedgehog (Shh) ligand modification by cholesterol [111]. Although the interaction of alcohol with the hedgehog pathway is well defined in certain conditions, more work needs to be performed on its involvement in CRC [109,110,111,112]. It can be inferred through more than one study that Shh signaling plays a role in angiogenesis in CRC, as angiogenesis is inhibited when Shh signaling is suppressed [113,114].

## 6. The Tumor Microenvironment

A dysregulated immune response provides a permissive microenvironment that helps tumor growth [13,115]. With regard to CRC, chronic inflammation plays a critical role in CRC development [116]. Chronic alcohol intake can cause mucosal inflammation in the intestine [5]. One mechanism that could induce an inflammatory response in the intestinal tract is via an increased exposure to lipopolysaccharide (LPS) from the intestinal microbiota [5]. The LPS production and exposure can be affected by ethanol, which is shown to induce microbial dysbiosis and bacterial overgrowth [5,117]. Ethanol, through multiple mechanisms, is also able to increase the permeability of the intestinal barrier (often referred to as “leaky gut”) that allows further LPS penetration and exposure [62,118]. Oxidative stress from the CYP2E1 metabolism of ethanol could increase intestinal permeability [119,120]. We have shown that, in a Caco-2 cell model, increased oxidative stress leads to increased CREB phosphorylation [119]. This CREB phosphorylation increases hyperpermeability, most likely, through increased ROS production, which can directly affect the barrier’s integrity [5]. ROS can also activate the EMT factor, snail [118]. Increased ROS production from acetaldehyde treatment in a Caco-2 cell model led to increased snail phosphorylation which, in turn, resulted in the redistribution of ZO-1, E-cadherin, and β-catenin [118]. This redistribution decreased the barrier function of the cell monolayer [118]. Our recent data suggests that colonic hyperpermeability from alcohol is at least partly mediated via alcohol’s epigenetic effects on Notch1 via altered histone H3 deacetylation at the Notch1 locus [121]. Besides ROS production, ethanol itself can affect cellular β-catenin distribution [122,123]. It has been shown in a HCT116 cell model that ethanol inactivates GSK3β, leading to an increased nuclear translocation of β-catenin [122]. This not only could contribute to cancer formation, but also to tumor aggression via production of MCP-1/CCR-2, and induction of cancer stem cell (CSC) metastasis [122]. Increased permeability and exposure to LPS and other microbial byproducts is sensed by immune cells, leading to the expression of pro-inflammatory cytokines like IL-6 and IL-18, as well as further release of ROS, fueling the vicious cycle [5,124]. Barrier dysfunction and increased LPS exposure from alcohol could shift toward a pro-tumorigenic inflammatory response [5,124]. For example, alcohol exposure in mice can lead to an increase in M2b macrophage recruitment [125]. This increase in M2 macrophages is significant, because such an increase has been associated with a poor prognosis in colon cancer patients [126].

There are other mechanisms that can contribute to ethanol’s increase in intestinal inflammation in cases of CRC [117,119,120,127,128,129]. Our group showed that chronic alcohol consumption accelerates polyposis in mice; the enhanced polyposis and submucosal invasion in our model was associated with an increased mast cell infiltration in the submucosa [13]. More recently, chronic ethanol feeding in an azoxymethane/dextran sulfate sodium (AOM/DSS) model of CRC in mice was shown to increase cell proliferation, and tumorigenesis [130]. The authors found that alcohol induced release of pro-tumorigenic inflammatory cytokines, such as IL-1α, IL-6, and TNFα, was associated with an enhanced immune cell infiltration and colonic inflammation in their model [130]. As intestinal inflammation is one of the main mechanisms via which alcohol promotes CRC, the interaction of alcohol with another factor that could induce intestinal inflammation is plausible.

We showed exacerbation of intestinal inflammation from alcohol by disruption of circadian rhythms, another common habit associated with modern lifestyle [131,132,133]. Recently, we have reported such an interaction in a CRC model in mouse. In a TS4Cre × APC^low468^ mouse model, we have shown that a diet including alcohol and a weekly 12 h phase reversal of light–dark cycles resulted in increased polyposis, along with an increased ratio of mMCP6+ mast cells to mMCP2+ mast cells in the intestinal tissue [128]. In the same set of mice, we also found a shift in the microbiota of the alcohol fed, circadian shifted mice where there was a decrease in the *Firmicutes*/*Bacteroidetes* ratio, correlating with decreased SCFA production [128]. These results suggest an interaction of circadian disruption and alcohol in CRC, potentially via promotion of a pro-tumorigenic inflammatory milieu.

## 7. Conclusions

**Figure 3:**
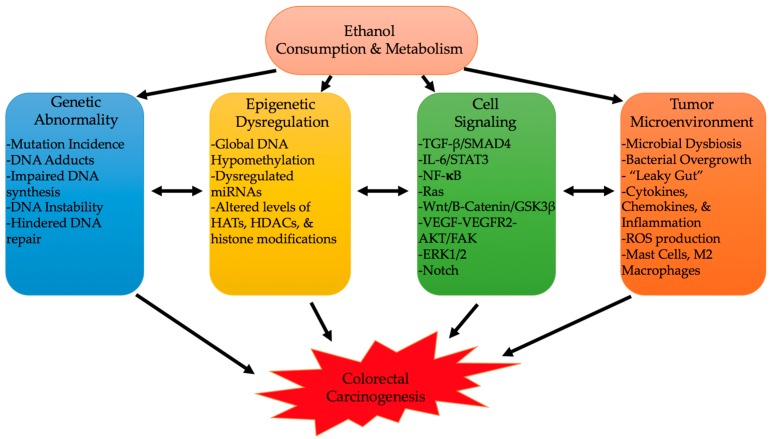
Genetic abnormality, epigenetic dysregulation, cell signaling, and the tumor microenvironment are the hallmark effects that ethanol produces to instigate colorectal carcinogenesis. These hallmark mechanisms interact and lead into each other to further increase the carcinogenic state.

The onset of CRC can be heavily influenced by environmental and lifestyle factors [4]. Alcohol consumption, a growing lifestyle habit in developed and some developing countries, appears to be one of the biggest CRC instigators [134,135,136,137]. Alcohol’s consumption and metabolism can have multiple molecular consequences that can instigate colon carcinogenesis [8,9,10]. Its oxidative and non-oxidative metabolism, and formation of byproducts, such as ROS and metabolites, can lead to a constellation of genetic, epigenetic, cell signaling, and immune processes (Figure 3) [5,9,10,12,55,65,66,67]. Interaction of these various mechanisms can affect cancer characteristics like proliferation, angiogenesis, stemness, EMT, oncogenic expression, and altered immunity [9,89,103,125]. Still, further work needs to be conducted to elucidate targetable driving mechanisms to prevent alcohol-induced CRC; the identification of such pathways can also help understand how other lifestyle factors interact with alcohol, and with each other, to promote CRC.
