# Correction: Rossi et al. Colorectal Cancer and Alcohol Consumption—Populations to Molecules. *Cancers* 2018, *10*, 38

**DOI:** 10.3390/cancers16233999

**Published:** 2024-11-29

**Authors:** Marco Rossi, Muhammad Jahanzaib Anwar, Ahmad Usman, Ali Keshavarzian, Faraz Bishehsari

**Affiliations:** Division of Digestive Diseases, Hepatology, and Nutrition, Department of Internal Medicine, Rush University Medical Center, Chicago, IL 60612, USA; marco_rossi@rush.edu (M.R.); docjazzya@gmail.com (M.J.A.); usman_ahmad@rush.edu (A.U.); ali_keshavarzian@rush.edu (A.K.)

There was a mistake in the introduction section as published [1]. The corrected sentence should be “It is estimated that alcohol increases the chance of CRC by more than 50% [7]”. 

There were errors in reference citations. The corrected references appear as follows:

7. Fedirko, V.; Tramacere, I.; Bagnardi, V.; Rota, M.; Scotti, L.; Islami, F.; Negri, E.; Straif, K.; Romieu, I.; La Vecchia, C.; et al. Alcohol drinking and colorectal cancer risk: An overall and dose-response meta-analysis of published studies. *Ann. Oncol.* **2011**, *22*, 1958–1972. https://doi.org/10.1093/annonc/mdq653.
16. Yi, S.W.; Sull, J.W.; Linton, J.A.; Nam, C.M.; Ohrr, H. Alcohol consumption and digestive cancer mortality in Koreans: The Kangwha Cohort Study. *J. Epidemiol.* **2010**, *20*, 204–211. https://doi.org/10.2188/jea.je20090077.

The authors state that the scientific conclusions are unaffected. This correction was approved by the Academic Editor. The original publication has also been updated.
